# Intensive physical training induces NET release in athletes

**DOI:** 10.1038/s41598-025-22886-3

**Published:** 2025-11-06

**Authors:** Joanna Orysiak, Jitendra Kumar Tripathi, Klaudia Brodaczewska, Atul Sharma, Konrad Witek, Jadwiga Malczewska-Lenczowska

**Affiliations:** 1 Central Institute for Labour Protection-National Research Institute, Warsaw, Poland; 2 Translational Pulmonary and Immunology Research Center , Long Beach, USA; 3 Military Institute of Medicine-National Research Institute, Warsaw, Poland; 4 The University of Texas MD Anderson Cancer Center , Houston, USA; 5 Institute of Sport-National Research Institute , Warsaw, Poland

**Keywords:** Innate immunity, Intensive training, Neutrophils, Muscle damage, Inflammatory markers, Ice hockey players, Biochemistry, Health care

## Abstract

The aim of this study was to determine the effect of intense physical training on the release of neutrophil extracellular traps (NETs) in URTI-free and URTI-prone athletes. Sixteen young male ice hockey players were monitored during a national training camp. Blood collection took place at the beginning and after the training camp. Blood tests included creatine kinase activity; white blood cell count and subpopulations; cortisol, immunoglobulin A and C-reactive protein concentrations; and the percentage of neutrophils releasing NETs. The occurrence of upper respiratory tract infections (URTI) was monitored using a validated URTI symptom questionnaire. There were no differences in immune and endocrine markers between URTI-free and URTI-prone athletes. Regardless of the subject’s health status, CK activity and uric acid levels increased significantly between the beginning and after the training camp. In both URTI-free and URTI-prone athletes, the percentage of neutrophils releasing NETs increased significantly after the training camp. Regardless of the athlete’s health status, a short period of intensive physical training itself may cause increased NET release in ice hockey players. The presence of mild URTI did not appear to be an additional factor influencing NET release during the several-day training camp.

**Supplementary Information:**

The online version contains supplementary material available at 10.1038/s41598-025-22886-3.

## Introduction

 Respiratory tract infections are responsible for approximately 50% of all illness episodes in athletes^1–4^, especially upper respiratory tract infections (URTI)^4,5^. URTI negatively impact athletes because they can disrupt training and competition by affecting the body’s normal response to training and/or affecting the body’s adaptation to regular training^4^.

Physical exercise has a transient impact on various immune parameters, including leukocyte (WBC, white blood cells) numbers and functions^6^. Intensive physical exercise induces biphasic mobilization of leukocyte subsets, which is characterized by initial neutrophilia, lymphocytosis, and monocytosis, followed by a delayed response of neutrophilia and lymphopenia^6,7^. The initial increase in neutrophils is likely due to demargination caused by shear stress and catecholamines, whereas the secondary elevation may be due to cortisol-induced release of neutrophils from bone marrow^7^. High exercise loads result in temporary suppression of the functioning of immune cells in athletes^8^. This suppression may make athletes more susceptible to pathogens causing upper respiratory tract infections, based on the “open window” theory^8^. Each bout of prolonged and intensive exercise has transient but significant, wide-ranging effects on the immune system^9^. If exercise is repeated in this immune-depressed state, this could lead to greater immunodepression and potentially a longer window of opportunity for infections^10^.

Neutrophils are one of the most important innate immune response components. They use three methods to kill pathogens: phagocytosis, degranulation, and neutrophil extracellular traps (NETs)^11^. An extracellular trap is composed of thin chromatin fibers with neutrophil proteins and enzymes (for example, neutrophil elastase, defensins, cathelicidin, myeloperoxidase, cathepsin G, and histones)^11^. A variety of stimuli may induce NETs—lipopolysaccharide, cytokines, reactive oxygen species (ROS), immunoglobulins, bacteria, viruses or protozoa^12–17^. One of the immunoglobulins that can enhance the release of NETs is immunoglobulin A (IgA)^13–15^. Human neutrophils may be activated by it, as they also express the Fc receptor (FcR) for IgA—FcαRI^15^. NETs have been posited to play a role in antibacterial immunity or inflammatory responses^12,18^. However, further research is needed on the role of neutrophils and NETs in viral infections in the upper respiratory tract^12,14,19^.

Physical exercise can also contribute to muscle damage and inflammatory responses^20,21^. Therefore, physical exercise also may enhance the release of NETs^18,22,23^. Short, acute physical exercise^22,23^, as well as longer intensive training, can increase the release of NETs^18^. It has been suggested that NET release during physical exercise may be one of the mechanisms that maintain immune homeostasis and prevent the development of chronic inflammation. However, excessive release of NETs or their improper removal, together with disturbances in other neutrophil functions, may lead not only to increased incidence of respiratory infections and chronic inflammation, but also to tissue/muscle damage and fatigue/overtraining^24^.

According to our knowledge, the relationship between NETs, intense physical training, and URTI remains poorly understood. Physical exercise may increase the release of NETs in healthy individuals, and during illness the amount of NET may also increase; therefore, we hypothesize that intensive physical training increases the percentage of NET-forming neutrophils to a greater extent in athletes with URTI symptoms compared to healthy ones. Due to the fact that athletes often train with mild URTI symptoms^4,25^, the aim of this study was to determine the effect of intense physical training on NET release in athletes with mild symptoms of URTI and in healthy representatives of the same sport discipline.

## Materials and methods

### Subjects

Sixteen young, elite/international level male ice hockey players (age 18.3 ± 0.6 years, height 181.2 ± 5.8 cm, body mass 80.5 ± 6.9 kg) were recruited during a national team training camp^26^. The study was conducted in accordance with the Declaration of Helsinki and approved by the Local Ethics Committee of the Institute Of Sport-National Research Institute (protocol code KEBN-18–37-JO). Written informed consent was obtained from athletes or their parents. This study includes some data previously presented in a pilot study^18^.

## Study design

**Fig. 1:**
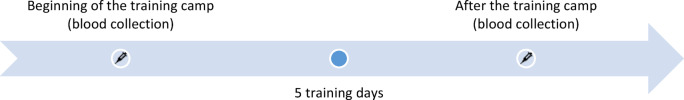
Blood collection scheme.

The national team training camp lasted 5 training days. This phase of training was characterized by high-training loads focusing on the development of physical fitness^18^ (about 12 h training on ice; about 6 h functional training and gym). Biological material (blood) was collected at the beginning of the training camp (before the start of training sessions) and after the training camp (after the end of the training sessions) (Fig. 1).

### Upper respiratory tract infections

The occurrence of upper respiratory tract infections was monitored using the validated URTI symptom questionnaire^27^ completed by athletes throughout the study. The questionnaire included a list of symptoms: sneezing, headache, malaise, nasal discharge, nasal obstruction, sore throat, cough, ear ache, hoarseness, fever, chilliness and joint aches and pains. Athletes with symptoms of infection were classified as “URTI-prone athletes”, while athletes without symptoms of infection were assigned to the “URTI-free athletes” group.

### Blood markers

Blood was taken from the cubital vein in the morning (between 7 and 9 am) after overnight fasting and a minimum of 12 h after the last training session. Haematological parameters were measured in whole blood collected into EDTA tubes. Blood samples for C-reactive protein (CRP), immunoglobulin A (IgA), creatine kinase (CK) and uric acid (UA), and cortisol measurements were collected into tubes containing coagulation accelerator and serum separator. To obtain serum for testing, the tubes with samples were centrifuged for 10 min at a speed of 2000 × g^18^.

White blood cells (WBC) and their subpopulations were measured using a hematology analyzer (XN 1000, Sysmex, Japan). C-reactive protein (CRP), immunoglobulin A (IgA), creatine kinase (CK) and uric acid (UA) were quantified in serum using the immunoturbidimetric method (for CRP and IgA) or the colorimetric method (for CK and UA) with a biochemical analyzer (Cobas Integra 400, Roche, Switzerland)^18^. An enzyme-linked immunoassay (ELISA) kit (DRG, Germany) was used to determine serum cortisol concentration^18^. All assays were conducted in the laboratory of the Department of Biochemistry, Institute of Sport— National Research Institute, under the implemented quality management system.

NET quantification was performed using the microscopy method. Neutrophils from human whole blood were isolated using the EasySep Direct Human Neutrophil Isolation Kit (STEM CELL, Germany) according to the manufacturer’s instructions. They were then cytocentrifuged onto glass slides and fixed with paraformaldehyde. The NET-forming neutrophils were visualized by staining the purified neutrophil populations with the highly sensitive nuclear stain Sytox Green. This allowed clear differentiation between NET-forming and non-NET-forming neutrophils under high-resolution fluorescence microscopy, based on the intact polymorphic nucleus vs. decondensed nuclear structure releasing DNA fibers. The percentage of neutrophils releasing NET was determined manually by dividing the number of NET-forming neutrophils by the total number of cells in 6 microscopic fields (3 where the number of NETs was the highest and 3 where the number of NETs was the lowest) and multiplying the values by 100. The detailed procedure has been described previously^18,28,29^.

### Rating of perceived exertion (RPE)

The intensity of the training camp was determined using the CR-10 Rating of Perceived Exertion (RPE) scale, ranging from “0” (rest) to “10” (maximal)^30^.

### Statistical analysis

 All markers were expressed as mean ± SD. The Shapiro–Wilk test and equal distribution test were used to verify the distribution of the data. Additionally, Cohen’s effect size was calculated^31^. The Wilcoxon signed-rank test or multiple paired t-tests, depending on the distribution, were used to evaluate the impact of training on the examined markers in athletes, separately for healthy ones (no symptoms of infection; URTI-free athletes; healthy) and those with URTI symptoms (URTI-prone athletes; URTI). The immune and endocrine markers were compared between the two groups of athletes using the nonparametric Mann–Whitney test or multiple unpaired t-test, as appropriate. Spearman’s or Pearson’s correlation coefficient was used to determine associations between IgA and NET. The level of statistical significance was set at *p* < 0.05. Detailed statistics are presented in Supplementary Information (Tables S1-S3; Figures S1-S4).

## Results

### Upper respiratory tract infections (URTI)

In this study 6 athletes had sneezing, malaise, nasal discharge, nasal obstruction, cough, hoarseness, fever as well as joint aches and pains on a scale from 1 (mild) to 2 (moderate) from the first to the last day of the training camp.

### URTI-prone athletes vs. URTI-free athletes

At the beginning of the training camp, significantly higher creatine kinase activity was observed in URTI-free athletes than in URTI-prone athletes (Table 1). There were no differences in immune and endocrine markers between the two groups of athletes at the beginning or after the training camp.

### Beginning vs. after training camp

**Fig. 2:**
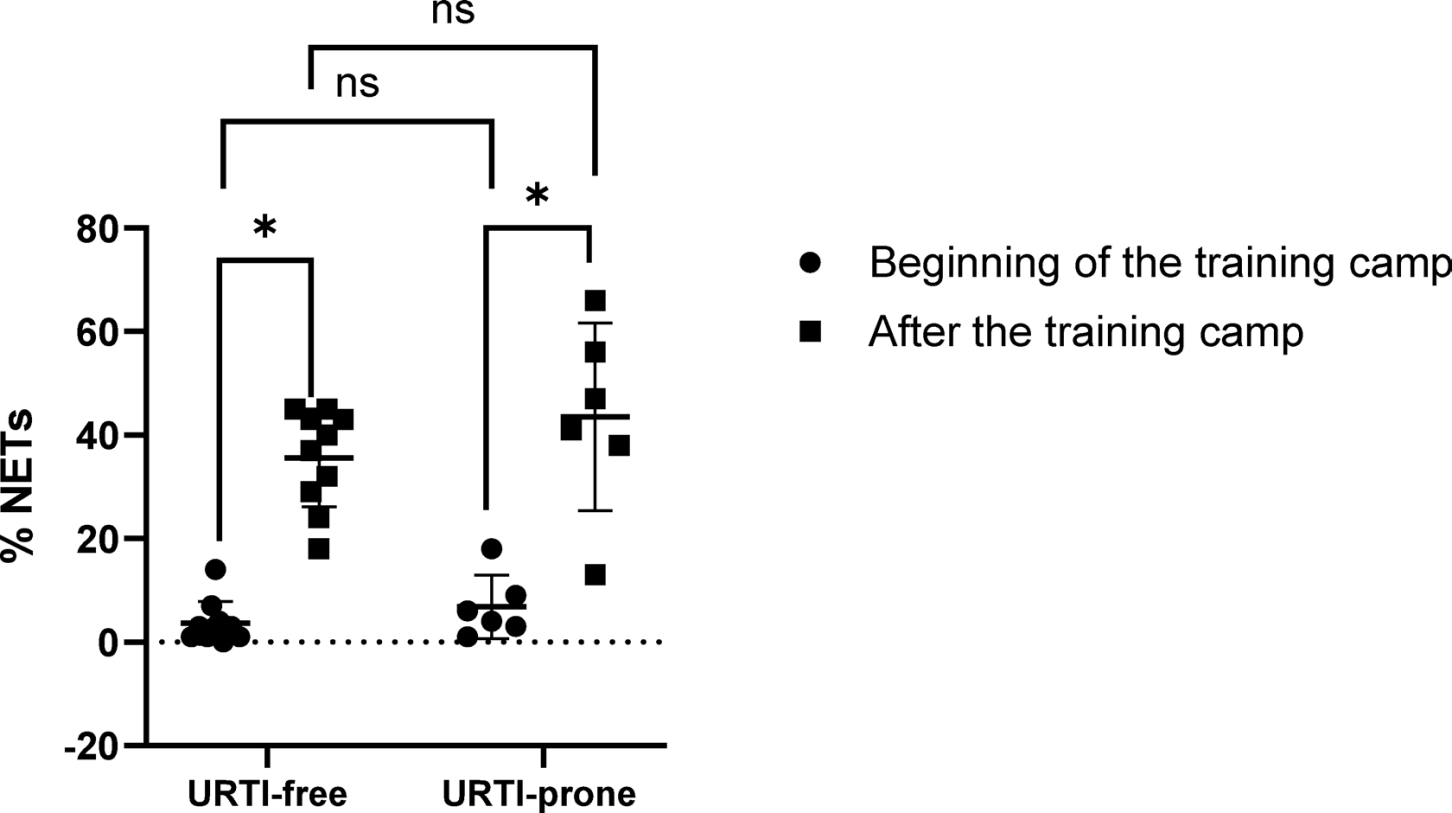
Effect of the training on the percentage of NET-forming neutrophils in studied groups. * *p* value < 0.05, ns – not significant. Individual values and mean ± SD are presented in the graph.

CK activity and uric acid levels increased significantly after the training camp in both groups of athletes (Table 1). A decrease in the WBC and neutrophil counts were observed after the training camp in both URTI-free and URTI-prone athletes. On the other hand, the percentage of NET-forming neutrophils increased significantly after the training camp in both groups of athletes (Fig. 2).

### Intensity of training

**Fig. 3:**
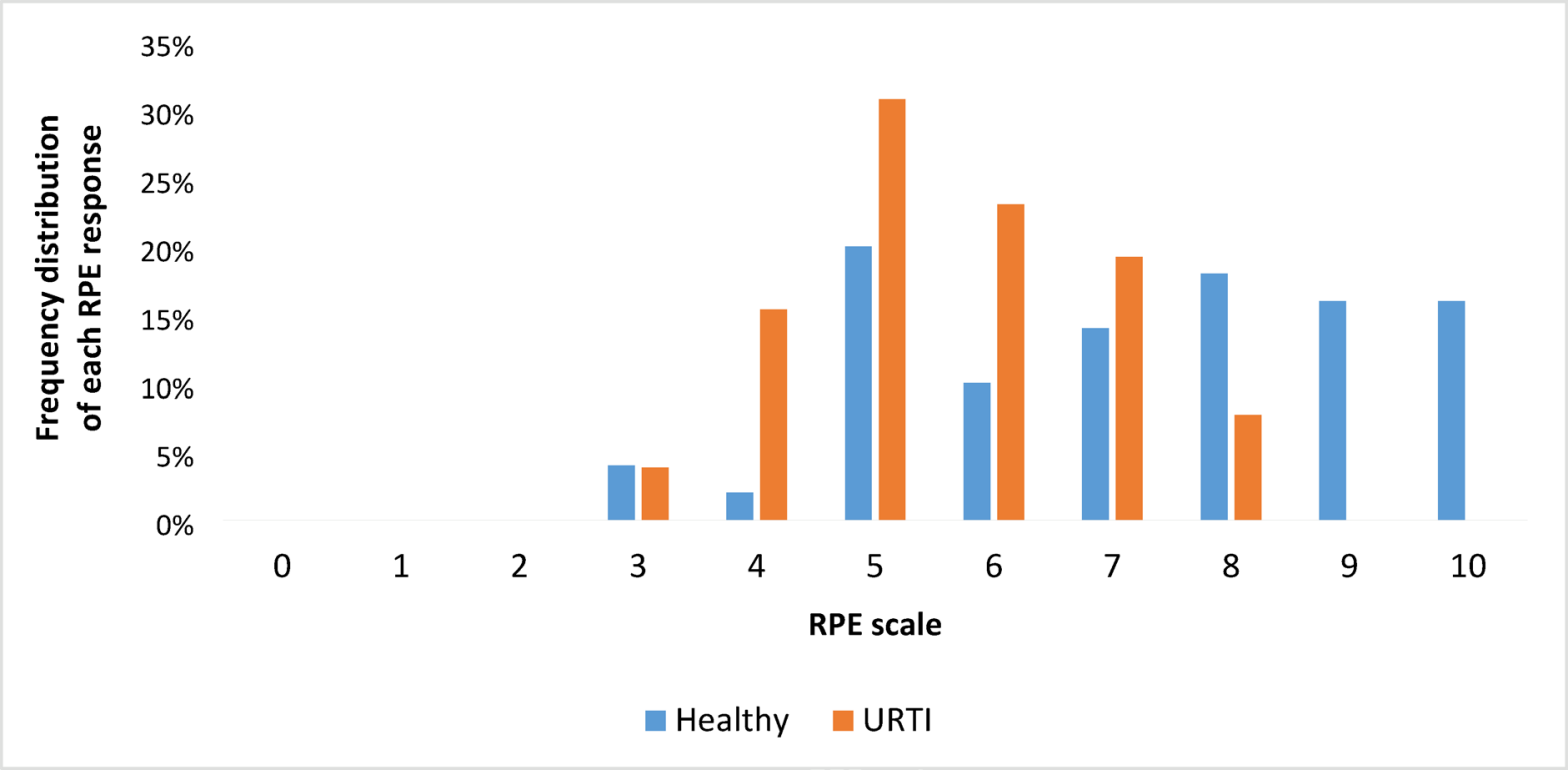
Frequency distribution of each RPE response in URTI-free and URTI-prone athletes.

The mean perceived intensity of the training camp was 7 ± 2 in URTI-free athletes and 6 ± 2 in URTI-prone athletes. The frequency distribution of each RPE response is shown in Fig. 3. The most frequently chosen answer in both groups was “5”.

### Immunoglobulin A vs. NET release

There was no significant association between NET release and IgA concentration in both groups of athletes. However, in the URTI-prone group, after the training camp the association was negative (*r*= −0.6, *p*-value = 0.2, compared to *r*= −0.28, *p*-value = 0.58 at the beginning).

**Table 1:** Descriptive means for blood markers analyzed at each time point (healthy *n* = 10; with URTI symptoms *n* = 6).

	URTI-free athletes (without URTI symptoms; HEALTHY)		URTI-prone athletes (with URTI symptoms; URTI)					
Markers	Beginning of the training camp (1)	After the training camp (2)	Beginning of the training camp (1)	After the training camp (2)	Difference p-values			
	Mean ± SD	Mean ± SD	Mean ± SD	Mean ± SD	Healthy1 vs. Healthy2	URTI1 vs. URTI2	Healthy 1 vs. URTI 1	Healthy 2 vs. URTI 2
Creatine kinase (U·L^−1^)	217 ± 99	584 ± 234	113 ± 31	832 ± 952	**0.002**	**0.031**	**0.022**	0.713
Uric Acid (µmol·L^−1^)	368 ± 74	423 ± 68	324 ± 42	427 ± 52	**0.005**	**0.003**	0.201	0.921
C-reactive protein (ng·dl^−1^)	0.6 ± 0.9	0.7 ± 0.9	0.3 ± 0.2	0.5 ± 0.5	**0.016**	0.081	0.251	0.409
Immunoglobulin A (g/l)	1.64 ± 0.89	1.51 ± 0.79	1.63 ± 0.46	1.56 ± 0.39	**0.002**	0.147	0.509	0.368
White blood cell (10^9^·L^−1^)	6.90 ± 1.38	6.01 ± 1.38	6.13 ± 1.24	4.79 ± 0.86	**0.020**	**0.031**	0.283	0.073
Neutrophil count (10^9^·L^−1^)	3.21 ± 0.84	2.63 ± 0.86	3.11 ± 0.79	2.09 ± 0.55	**0.013**	**0.024**	0.805	0.193
Lymphocyte count (10^9^·L^−1^)	2.76 ± 0.65	2.57 ± 0.48	2.22 ± 0.36	2.11 ± 0.34	0.242	0.240	0.057	0.061
Monocyte count (10^9^·L^−1^)	0.59 ± 0.15	0.55 ± 0.18	0.54 ± 0.16	0.42 ± 0.06	0.262	0.094	0.559	0.208
Eosinophil count (10^9^·L^−1^)	0.24 ± 0.20	0.20 ± 0.15	0.16 ± 0.06	0.11 ± 0.04	0.120	**0.031**	0.792	0.474
Basophil count (10^9^·L^−1^)	0.04 ± 0.02	0.04 ± 0.02	0.04 ± 0.02	0.04 ± 0.01	0.563	0.750	0.836	0.385
NET (%)	4 ± 4	36 ± 9	7 ± 6	44 ± 18	**0.002**	**0.031**	0.183	0.267
Cortisol (nmol·L^−1^)	731 ± 199	693 ± 227	591 ± 190	744 ± 207	0.428	0.092	0.187	0.661

Significant outcomes are highlighted in bold font *p* < 0.05.

## Discussion

To the best of our knowledge, no studies have examined the percentage of NET-forming neutrophils in athletes with respiratory tract infection symptoms during a training camp. In this study, after a short, 5-day period of intensive training, CK activity, uric acid concentration, and percentage of NET-forming neutrophils increased in both URTI-free and URTI-prone athletes. However, there were no significant differences between URTI-free and URTI-prone athletes. These results thus suggested that intense physical exercise induces a greater percentage of NET-forming neutrophils than mild URTI .

Viruses may induce NETs^12,16,32–34^. NETs play an important role in defending against viral infections by preventing virus spread by trapping and immobilizing viral particles^12,34^. Moreover, NETs can increase the local concentration of antimicrobial peptides (for example, cathelicidins or defensins), which play an important role in the antiviral response^12,34^. On the other hand, excessive or unnecessary NET release may lead to tissue damage^12^. Therefore, tight control of NET release is needed to avoid NET-induced pathogenesis^12^. Elevated levels of cell-free DNA (cfDNA), myeloperoxidase-DNA (MPO-DNA), and citrullinated histone H3 (Cit-H3) were observed in sera from patients with COVID-19^33^. Moreover, patients with severe influenza A virus infection had elevated plasma levels of NETs, measured by cell-free DNA level and DNA-MPO complexes, and high NET release correlated with lung injury and influenza severity^32^. In our study, there were no statistically significant differences in the percentage of neutrophils releasing NETs between URTI-prone athletes and URTI-free athletes. Previous studies have shown that serious respiratory viral infections, such as influenza, COVID-19, and human respiratory syncytial virus (hRSV), increase NET release^16,32,33,35^. In ice hockey players the lack of significant differences in levels of tested markers between groups may suggest very mild URTI in URTI-prone athletes. Due to only mild or moderate symptoms of URTI in athletes, we may not have observed statistically significant differences in the percentage of neutrophils releasing NETs between athlete groups. Therefore, the severity of the infection may have influenced the results^16,34,35^.

Immunoglobulin A is another factor that can induce NET release^14,16^. In the serum there are two subtypes of IgA—IgA1 and IgA2 (their proportions in the serum are 90% and 10%, respectively). It has been reported that IgA2 has a pro-inflammatory effect on neutrophils and macrophages and that it induces NET release^36,37^. Moreover, IgA-virus immune complexes may potentiate NET release^14^. However, in our study, there was no association between IgA concentration and NET release. Furthermore, the IgA level in URTI-free athletes decreased after training, but NETs were still induced at this time point, suggesting that NET release after physical exercise was likely induced by other factors. However, in the URTI-prone group, where a concurrent pathogen insult may have been present, we did not observe an elevated IgA concentration compared to the healthy group, either at the beginning or after the observation period. Previous studies also have not identified a relationship between serum IgA concentration and upper respiratory tract infections^38–40^. Lower IgA concentrations were observed in individuals with community-acquired pneumonia (CAP) in comparison to healthy persons^41^. In turn, IgA concentration was significantly higher in the COVID-19 pneumonia group than the ambulatory group, and a high concentration in the acute phase of COVID-19 increased the risk of pneumonia 3.4-fold^42^. It is possible that in the ice hockey players, the URTI was too mild to increase the level of IgA in the serum.

In sport science, numerous blood markers reflect physiological alterations (for example, muscle damage or inflammation) occurring after intensive physical exercise. The most commonly used are lactate, CK, CRP, urea, and WBC^43–46^. Creatine kinase is a marker of muscle damage and training load^47^. Cortisol is known to be a marker of stress and training load over a longer period than CK. Therefore, it can be used to check for any deeper signs of overload that might not be detected by CK^48^. Lactic acid produced during high-intensity exercise inhibits UA excretion, leading to elevated UA levels; therefore, UA concentration can be used as an additional training load marker^49^. In our study, an increase in creatine kinase activity and uric acid levels after the training camp was observed in both URTI-free and URTI-prone athletes. These results confirm the occurrence of muscle damage in athletes subjected to heavy training loads. Additionally, a decrease in WBC counts and neutrophil counts was observed after the training camp. The main sign of exercise-induced muscle damage (EIMD) is the accumulation of immune cells in the muscle tissue^21^. For this reason it cannot be ruled out that the decrease in neutrophils may represent an aspect of inflammation due to increased migration and accumulation of these cells in muscle-damaged tissue caused by intensive physical training^18,50,51^.

Physical exercise can also be a factor inducing NET release, but the amount of NETs depends on the type, duration, and intensity of physical exercise. Previous studies have shown that acute or short, intense physical exercise causes NET release^22,23,52^. In contrast, long-term physical exercise (several weeks) did not increase NET release^53,54^. In our study, 9-fold and 6-fold increases in NET-forming neutrophils were observed in URTI-free athletes and URTI-prone athletes, respectively. These results confirmed that intense, multi-day physical exercise causes NET release regardless of the athlete’s health status, which is also consistent with our previous research^18^.

In this study, there were no differences in certain markers of the immune system’s response to intense physical training between URTI-free and URTI-prone athletes. It is worth noting that athletes with symptoms of infection often do not give up training^25^, but the long-term consequences of training with infection remain largely unknown^4^. Infection can affect, various factors including rating of perceived exertion, physical performance during training or competition, ability to train (reduced training load), and the occurrence of injuries^4,55,56^. However, it has been observed that the effect on physical performance depends on the severity of symptoms: mild symptoms are associated with a relatively low risk of a negative impact compared to moderate-severe symptoms^4,55,57^.

In a study by Weider et al.^58^, 40 min of moderate training in individuals with symptoms of infection did not prolong or worsen the illness symptoms. Therefore, it was suggested that if symptoms occur “above the neck,” low-intensity training may be safe^59^. Infections such as SARS-CoV-2, adenovirus, enterovirus or influenza virus may pose a risk to the circulatory system, and in other, milder cases, athletes may train, but the decision regarding training should be personalized and made after medical consultation^60^. However, it should be remembered that symptoms can change over time and worsen, and that intensive training can lead to various complications, such as chronic fatigue or myocarditis^25,59,61^. It is often difficult for athletes to refrain from training when ill, especially in team sports, due to pressure not to “leave the team short”^59^. However, because it is challenging to determine safe training loads for ill athletes (and whether the training staff and the athlete will adhere to them), in competitive sports, ill athletes should generally refrain from training^59^. Athletes and coaching/training staff should also remember that training with an infection promotes the spread of the virus to other teammates, which can weaken the team during an important match or competition^59,62^.

Regarding limitations, the small sample size (*n* = 6) of ill athletes could be problematic, although only these athletes exhibited URTI symptoms during the training camp. Generally, it is difficult to recruit a large cohort of athletes who will develop URTI simultaneously and under the same training load. For this reason, ice hockey was selected for the study, as it is a discipline trained in conditions favorable for infections and/or injury^40,63^. In addition to biochemical indicators, it would be valuable to measure exercise indicators, such as loss of muscle strength and power, to assess muscle damage^21^. Future studies should also include women, as the immune system’s response to physical exertion may vary by sex. Moreover, examining other functions of neutrophils would allow for a more accurate assessment of immune system functioning. The lack of monitoring of food and dietary supplement consumption can also be considered a limitation. However, all athletes ate in the same canteen with a limited choice of food. Additionally, in the surveys they did not report regular use of specific medications, or dietary or nutritional supplements. The fact that the study investigated specialized athletes from the national team - some of the best players in this discipline in the country - can be considered both a strength and a limitation, as the results apply only to this highly specific group. Another strength of the research is that it was conducted during a national team training camp, ensuring real and identical training conditions. Moreover, direct methods of quantification of NETs by immunofluorescence microscopy are more appropriate and objective than indirect methods, such as using cfDNA, which can originate not only from NET released during NETosis but also from apoptotic or necrotic cells^64–66^.

## Conclusions

The results indicate that regardless health status, a short period of intensive physical training itself may cause increased NET release in ice hockey players.

The presence of mild URTI did not appear to be an additional factor influencing NET-forming neutrophils during the training camp. Nevertheless, it is still not recommended that athletes with URTI symptoms participate in intensive training.

## Supplementary Information

Below is the link to the electronic supplementary material.

Supplementary Material 1

## Data Availability

The datasets generated and/or analysed during the current study are not publicly available due to reasons of sensitivity but are available from the corresponding author on reasonable request.
