# Supplementary material for: Intensive physical training induces NET release in athletes
Source: Sci Rep. 2025 Nov 6;15:38983. doi: 10.1038/s41598-025-22886-3 (PMC12592488; doi:10.1038/s41598-025-22886-3)
Supplement: Supplementary file 1 — Supplementary Material 1 [file 41598_2025_22886_MOESM1_ESM.docx]

**Intensive physical training induces** **NET release** **in athletes**

Table S1 Summary of statistics performed.

| URTI-free | Beginning of the training camp | | | | | After the training camp | | | | |
| --- | --- | --- | --- | --- | --- | --- | --- | --- | --- | --- |
|  | mean | median | SD | SEM | N | mean | median | SD | SEM | N |
| IgA | 1.64 | 1.14 | 0.86 | 0.27 | 10 | 1.51 | 1.07 | 0.79 | 0.25 | 10 |
| NET | 3.70 | 3.00 | 4.14 | 1.31 | 10 | 35.60 | 38.50 | 9.48 | 3.00 | 10 |
| CK | 217.30 | 210.00 | 98.98 | 31.30 | 10 | 583.60 | 589.00 | 234.04 | 74.01 | 10 |
| Uric Acid | 367.90 | 378.00 | 73.67 | 23.30 | 10 | 423.37 | 428.90 | 67.52 | 21.35 | 10 |
| WBC | 6.90 | 6.62 | 1.38 | 0.44 | 10 | 6.01 | 5.97 | 1.38 | 0.44 | 10 |
| CRP | 0.59 | 0.40 | 0.66 | 0.21 | 10 | 0.86 | 0.55 | 0.93 | 0.29 | 10 |
| #NEUT | 3.21 | 3.25 | 0.84 | 0.26 | 10 | 2.63 | 2.55 | 0.86 | 0.27 | 10 |
| #LYMPH | 2.76 | 2.76 | 0.65 | 0.21 | 10 | 2.57 | 2.55 | 0.48 | 0.15 | 10 |
| #MONO | 0.59 | 0.56 | 0.15 | 0.05 | 10 | 0.55 | 0.51 | 0.18 | 0.06 | 10 |
| #EOS | 0.24 | 0.18 | 0.20 | 0.06 | 10 | 0.20 | 0.16 | 0.15 | 0.05 | 10 |
| #BASO | 0.04 | 0.04 | 0.02 | 0.01 | 10 | 0.04 | 0.05 | 0.02 | 0.01 | 10 |
| Cortisol | 731.20 | 708.50 | 199.17 | 62.98 | 10 | 693.30 | 627.50 | 227.34 | 71.89 | 10 |
|  |  |  |  |  |  |  |  |  |  |  |
| URTI-prone | Beginning of the training camp | | | | | After the training camp | | | | |
|  | mean | median | SD | SEM | N | mean | median | SD | SEM | N |
| IgA | 1.63 | 1.69 | 0.46 | 0.19 | 6 | 1.56 | 1.60 | 0.39 | 0.16 | 6 |
| NET | 6.83 | 5.00 | 6.11 | 2.50 | 6 | 43.50 | 44.00 | 18.12 | 7.40 | 6 |
| CK | 113.00 | 103.50 | 31.39 | 12.81 | 6 | 832.33 | 478.50 | 952.18 | 388.73 | 6 |
| Uric Acid | 323.50 | 328.00 | 41.61 | 16.99 | 6 | 426.58 | 423.70 | 51.81 | 21.15 | 6 |
| WBC | 6.13 | 6.38 | 1.24 | 0.51 | 6 | 4.79 | 4.78 | 0.86 | 0.35 | 6 |
| CRP | 0.27 | 0.30 | 0.23 | 0.10 | 6 | 0.52 | 0.45 | 0.46 | 0.19 | 6 |
| #NEUT | 3.11 | 3.17 | 0.79 | 0.32 | 6 | 2.09 | 2.06 | 0.55 | 0.22 | 6 |
| #LYMPH | 2.22 | 2.22 | 0.36 | 0.15 | 6 | 2.11 | 2.21 | 0.34 | 0.14 | 6 |
| #MONO | 0.54 | 0.52 | 0.16 | 0.07 | 6 | 0.42 | 0.44 | 0.06 | 0.02 | 6 |
| #EOS | 0.16 | 0.15 | 0.06 | 0.02 | 6 | 0.11 | 0.12 | 0.04 | 0.02 | 6 |
| #BASO | 0.04 | 0.03 | 0.02 | 0.01 | 6 | 0.04 | 0.03 | 0.01 | 0.00 | 6 |
| Cortisol | 591.00 | 634.50 | 189.67 | 77.43 | 6 | 744.33 | 823.00 | 207.09 | 84.55 | 6 |

Table S2 Summary of statistics performed.

|  | Passed normality for both groups? (Shapiro-Wilk) | P value for F test | Used test | P value | q value |  | Significant? |  | Cohen's effect size |
| --- | --- | --- | --- | --- | --- | --- | --- | --- | --- |
| Healthy 1 vs 2 |  |  |  |  |  |  |  |  |  |
| IgA | No |  | Wilcoxon | 0.0020 | 0.0059 |  | Yes |  | -0.16 |
| NET | No |  | Wilcoxon | 0.0020 | 0.0059 |  | Yes |  | 4.36 |
| CK | Yes | 0.0172 | Wilcoxon | 0.0020 | 0.0059 |  | Yes |  | 2.04 |
| Uric Acid | Yes | 0.7993 | T test | 0.0048 | 0.0128 |  | Yes |  | 0.78 |
| WBC | Yes | 0.9944 | T test | 0.0223 | 0.0376 |  | Yes |  | -0.64 |
| CRP | No |  | Wilcoxon | 0.0156 | 0.0254 |  | Yes |  | 0.34 |
| #NEUT | Yes | 0.9343 | T test | 0.0131 | 0.0265 |  | Yes |  | -0.69 |
| #LYMPH | Yes | 0.3901 | T test | 0.2429 | 0.2646 |  | No |  | -0.33 |
| #MONO | Yes | 0.5089 | T test | 0.2620 | 0.2646 |  | No |  | -0.26 |
| #EOS | Yes | 0.4795 | T test | 0.1200 | 0.1514 |  | No |  | -0.23 |
| #BASO | No |  | Wilcoxon | 0.5625 | 0.4261 |  | No |  | 0.11 |
| Cortisol | Yes | 0.6999 | T test | 0.4282 | 0.3931 |  | No |  | -0.18 |
|  |  |  |  |  |  |  |  |  |  |
| URTI 1 vs 2 |  |  |  |  |  |  |  |  |  |
| IgA | Yes | 0.7279 | T test | 0.1470 | 0.1782 |  | No |  | -0.15 |
| NET | Yes | 0.0324 | Wilcoxon | 0.0313 | 0.0631 |  | Yes |  | 2.71 |
| CK | No |  | Wilcoxon | 0.0313 | 0.0631 |  | Yes |  | 1.07 |
| Uric Acid | Yes | 0.6419 | T test | 0.0035 | 0.0374 |  | Yes |  | 2.19 |
| WBC | Yes | 0.4337 | T test | 0.0306 | 0.0741 |  | Yes |  | -1.25 |
| CRP | Yes | 0.1664 | T test | 0.0812 | 0.1453 |  | No |  | 0.69 |
| #NEUT | Yes | 0.4465 | T test | 0.0244 | 0.0738 |  | Yes |  | -1.50 |
| #LYMPH | Yes | 0.9100 | T test | 0.2402 | 0.2646 |  | No |  | -0.32 |
| #MONO | Yes | 0.0514 | T test | 0.0959 | 0.1453 |  | No |  | -1.00 |
| #EOS | Yes | 0.3916 | T test | 0.0093 | 0.0374 |  | Yes |  | -0.85 |
| #BASO | No |  | Wilcoxon | 0.7500 | 0.7575 |  | No |  | -0.26 |
| Cortisol | Yes | 0.8518 | T test | 0.0924 | 0.1453 |  | No |  | 0.77 |
|  |  |  |  |  |  |  |  |  |  |
| Healthy 1 vs URTI 1 | |  |  |  |  |  |  |  |  |
| IgA | No |  | Mann Whitney | 0.5091 | 0.7713 |  | No |  | -0.02 |
| NET | No |  | Mann Whitney | 0.1828 | 0.4431 |  | No |  | 0.60 |
| CK | Yes | 0.0210 | Mann Whitney | 0.0225 | 0.2724 |  | Yes |  | -1.42 |
| Uric Acid | Yes | 0.2211 | T test | 0.2011 | 0.4900 |  | No |  | -0.74 |
| WBC | Yes | 0.8589 | T test | 0.2830 | 0.4900 |  | No |  | -0.59 |
| CRP | No |  | Mann Whitney | 0.2511 | 0.5073 |  | No |  | -0.66 |
| #NEUT | Yes | 0.9444 | T test | 0.8058 | 0.8878 |  | No |  | -0.13 |
| #LYMPH | Yes | 0.2109 | T test | 0.0869 | 0.4900 |  | No |  | -1.02 |
| #MONO | Yes | 0.7336 | T test | 0.5594 | 0.7533 |  | No |  | -0.30 |
| #EOS | Yes | 0.0173 | Mann Whitney | 0.7921 | 0.8612 |  | No |  | -0.54 |
| #BASO | No |  | Mann Whitney | 0.6801 | 0.8367 |  | No |  | -0.14 |
| Cortisol | Yes | 0.9662 | T test | 0.1873 | 0.4900 |  | No |  | -0.72 |
|  |  |  |  |  |  |  |  |  |  |
| Healthy 2 vs URTI 2 | |  |  |  |  |  |  |  |  |
| IgA | No |  | Mann Whitney | 0.3676 | 0.6203 |  | No |  | 0.08 |
| NET | Yes | 0.0881 | T test | 0.2668 | 0.5276 |  | No |  | 0.55 |
| CK | No |  | Mann Whitney | 0.7128 | 0.7854 |  | No |  | 0.36 |
| Uric Acid | Yes | 0.5809 | T test | 0.9219 | 0.9312 |  | No |  | 0.05 |
| WBC | Yes | 0.3084 | T test | 0.0725 | 0.4394 |  | No |  | -1.06 |
| CRP | No |  | Mann Whitney | 0.4095 | 0.6203 |  | No |  | -0.47 |
| #NEUT | Yes | 0.3345 | T test | 0.1927 | 0.5276 |  | No |  | -0.75 |
| #LYMPH | Yes | 0.4682 | T test | 0.0614 | 0.4394 |  | No |  | -1.10 |
| #MONO | Yes | 0.0264 | Mann Whitney | 0.2078 | 0.6099 |  | No |  | -0.92 |
| #EOS | Yes | 0.0081 | Mann Whitney | 0.4744 | 0.6389 |  | No |  | -0.72 |
| #BASO | No |  | Mann Whitney | 0.3851 | 0.6203 |  | No |  | -0.60 |
| Cortisol | Yes | 0.8812 | T test | 0.6606 | 0.8007 |  | No |  | 0.23 |

Table S3 Correlation statistics for NET and IgA values

| Correlation between NETs and IgA | |  |  |  |
| --- | --- | --- | --- | --- |
|  | Normal ditribution? | Used test | r value | p value |
| Healthy 1 | No | Spearman | -0.1429 | 0.7018 |
| Healthy 2 | No | Spearman | -0.0427 | 0.9115 |
| URTI 1 | Yes | Pearson | 0.2875 | 0.5807 |
| URTI 2 | Yes | Pearson | -0.6000 | 0.2080 |

Figure S1 Effect of the training on the creatine kinase activity in studied groups. * *p* value < 0.05, ns – not significant. Individual values and mean ± SD are presented in the graph.

Figure S2 Effect of the training on the IgA concentration in studied groups. * *p* value < 0.05, ns – not significant. Individual values and mean ± SD are presented in the graph.

Figure S3 Effect of the training on the WBC counts in studied groups. * *p* value < 0.05, ns – not significant. Individual values and mean ± SD are presented in the graph.

Figure S4 Effect of the training on the neutrophil counts in studied groups. * *p* value < 0.05, ns – not significant. Individual values and mean ± SD are presented in the graph.
